# Protective Effects of Humic Acid on Intestinal Barrier Dysfunction and Inflammatory Activation in Canine Cell-Based Models

**DOI:** 10.3390/ani16020173

**Published:** 2026-01-07

**Authors:** Alma Virág Móritz, Orsolya Farkas, Ákos Jerzsele, Nikolett Palkovicsné Pézsa

**Affiliations:** 1 Department of Pharmacology and Toxicology, University of Veterinary Medicine Budapest, 1078 Budapest, Hungary; moritz.alma.virag@univet.hu (A.V.M.); farkas.orsolya@univet.hu (O.F.); jerzsele.akos@univet.hu (Á.J.); 2 National Laboratory of Infectious Animal Diseases, Antimicrobial Resistance, Veterinary Public Health and Food Chain Safety, University of Veterinary Medicine, 1078 Budapest, Hungary

**Keywords:** canine model, chronic enteropathy, humic acid, inflammation, intestinal barrier, IPEC-J2 cells, lipopolysaccharide (LPS), oxidative stress, PBMCs

## Abstract

The intestinal barrier is essential for gastrointestinal and systemic homeostasis by enabling nutrient absorption while limiting the translocation of pathogens and toxins. When barrier function is impaired, bacterial components such as lipopolysaccharides (LPSs) may cross the epithelium and promote inflammatory signaling. In dogs, chronic inflammatory enteropathies are frequent disorders associated with barrier dysfunction, dysbiosis, and immune dysregulation, and may progress to protein-losing enteropathy or systemic inflammation. Humic substances, particularly humic acid (HA), are natural organic compounds with reported antioxidative, immunomodulatory, and barrier-supporting effects; however, the cellular mechanisms underlying these effects in intestinal and immune models remain insufficiently characterized. This study evaluated the effects of a commercially available HA-based supplement on epithelial barrier integrity and inflammatory responses using an in vitro system combining IPEC-J2 intestinal epithelial cells and primary canine peripheral blood mononuclear cells (PBMCs). Epithelial barrier integrity (FD4 paracellular flux), reactive oxygen species, and cytokine production (TNF-α, IL-6) were assessed under basal and LPS-stimulated conditions. HA treatment preserved epithelial barrier function and reduced LPS-induced pro-inflammatory cytokine production, supporting further investigation of HA as a nutraceutical adjunct for gut health support in dogs with chronic enteropathies.

## Simple Summary

Chronic enteropathies in dogs are often linked to impaired intestinal barrier function and inflammation. When the barrier is weakened, bacterial components such as lipopolysaccharides (LPSs) can stimulate inflammatory responses. Humic substances are natural compounds that have been proposed to support gut health, but their cellular effects are not well defined. In this study, we used intestinal epithelial cells (IPEC-J2) and canine immune cells (PBMCs) to test a humic acid-based supplement. We assessed epithelial barrier integrity by measuring FITC-dextran (FD4) passage and assessed inflammation by measuring cytokines (TNF-α, IL-6). Humic acid (especially when sonicated) helped preserve epithelial barrier integrity and reduced pro-inflammatory cytokine production under LPS challenge. These results support further evaluation of humic substances as nutraceutical candidates to support gut barrier function and inflammatory control in dogs.

## 1. Introduction

The intestine acts as a crucial barrier that separates the contents of the intestinal lumen from the rest of the body and performs multiple roles simultaneously. These include limiting interactions with both commensal and pathogenic bacteria, detoxifying bacterial endotoxins, regulating nutrient absorption, restricting the passage of harmful substances and microbes, initiating immune responses, and preventing the overgrowth of pathogenic organisms [1,2,3]. It is made up of multiple layers that together provide a complete physical and functional barrier. Disruption of any component of the intestinal barrier can lead to its dysfunction; however, increased paracellular permeability resulting from tight junction (TJ) damage is regarded as the most critical factor [1]. The physical protection is primarily ensured by a single layer of confluent intestinal epithelial cells (enterocytes), which form a continuous cellular sheet sealed by specialized dynamic intercellular protein complexes known as TJ. These junctional structures are essential for maintaining epithelial cohesion and the barrier’s selective permeability. In addition to TJs, adherens junctions and gap junctions contribute mainly to mechanical adhesion and intercellular communication, respectively, but they do not directly regulate paracellular permeability. TJs effectively close the apical paracellular space, thereby controlling the intercellular transport of ions and small molecules while preventing the translocation of pathogens. The TJ complex is composed of multiple transmembrane and cytoplasmic proteins, among which the claudin family plays a central role in determining barrier selectivity and permeability characteristics. In contrast, the absorption of macromolecules such as nutrients generally occurs via the transcellular route rather than through the paracellular space. TJ integrity is therefore essential for maintaining epithelial homeostasis and the overall intestinal barrier function [2,3]. Dysregulation or disassembly of these complexes is strongly associated with various pathological conditions, including inflammatory bowel diseases and metabolic endotoxemia [4]. In the gastrointestinal tract, both host-derived inflammatory mediators and pathogenic microorganisms—such as *Salmonella* spp.—can disrupt TJ integrity, exacerbating mucosal inflammation and contributing to the progression of chronic intestinal disorders. When intestinal barrier dysfunction occurs, disruption of TJs allows luminal substances—such as bacterial lipopolysaccharide (LPS) and other microbial products—to pass between epithelial cells into the lamina propria and, eventually, into the systemic circulation. This translocation triggers local and systemic inflammatory responses, contributing to the development and progression of chronic inflammatory disorders [5,6]. A central player in this response is the activation of nuclear factor kappa-light-chain-enhancer of activated B cells (NF-κB). Once activated, NF-κB translocates to the nucleus and initiates the transcription of genes involved in immune and stress responses [7]. This leads to the production of proinflammatory cytokines, including tumor necrosis factor-alpha (TNF-α), interleukin-1β (IL-1β), and interleukin-6 (IL-6), which promote tissue inflammation. Such localized intestinal inflammation is a key factor in the development of various gastrointestinal diseases [1]. Once LPS enters the bloodstream it encounters immune cells within the intestinal mucosa, where it is recognized as a pathogen-associated molecular pattern (PAMP). This recognition triggers a robust inflammatory response, characterized by the activation of macrophages and neutrophils that generate large amounts of reactive oxygen species (ROS) as part of the host defense mechanism. While ROS production serves to eliminate invading pathogens, excessive or chronic activation—such as under conditions of sustained LPS exposure—can damage host cells and tissues, exacerbating inflammation and oxidative stress. Importantly, LPS itself does not directly produce ROS but rather induces immune cells to generate them in large quantities as a downstream consequence of immune activation [8,9].

The disruption of intestinal barrier integrity and the ensuing inflammatory and oxidative processes are key events implicated in the pathogenesis of several chronic gastrointestinal disorders, including canine inflammatory enteropathy (CIE). CIE in dogs refers to a group of idiopathic inflammatory bowel conditions, marked by ongoing gastrointestinal symptoms such as diarrhea, vomiting, and weight loss, along with histological evidence of inflammation in the intestinal tissue. CIE can be classified based on their response to treatment into four categories: food-responsive enteropathy (FRE), antibiotic-responsive enteropathy (ARE), immunosuppressant-responsive enteropathy (IRE), and non-responsive enteropathy (NRE). In addition to classification based on treatment response, dogs that lose protein through the gastrointestinal tract are typically categorized under protein-losing enteropathy (PLE) [10]. The IRE and NRE groups also encompass idiopathic inflammatory bowel disease (IBD), a condition of unknown origin, which can only be diagnosed through histopathological examination of the intestinal mucosa [11]. IBD is linked to oxidative stress, with inflammatory cytokines released during the condition promoting increased production of ROS and further inflammation [10]. Additionally, the disease may elevate the risk of endotoxemia triggered by LPS [12,13]. The pathogenesis of CIE involves a complex interaction between genetic susceptibility, impaired intestinal barrier function, microbial imbalance (dysbiosis), and abnormal immune responses [14]. Epidemiological data indicate that CIE affects approximately 10–15% of dogs with persistent gastrointestinal symptoms. Severe forms, such as PLE, are particularly prevalent in certain breeds, including Soft Coated Wheaten Terriers and Yorkshire Terriers, and are associated with a poor prognosis due to complications like hypoalbuminemia, thromboembolism, and systemic illness [13,15]. Current treatment strategies—spanning dietary trials to immunosuppressive therapy—remain limited in effectiveness, highlighting the urgent need for innovative approaches that address both the gut microbiota and mucosal barrier integrity [15]. Despite their widespread use, these interventions often provide only partial or temporary relief, and long-term management remains challenging due to recurrent inflammation and treatment-associated side effects. Given these limitations, there is growing interest in natural adjunct therapies that can support intestinal health, reduce reliance on antibiotics, and potentially minimize the required doses of immunosuppressive drugs.

Humic substances (HS)—comprising humic acid (HA), fulvic acid (FA), and humin—are complex, heterogeneous supramolecular structures formed through the microbial and chemical degradation of plant and animal residues in soil, peat, and aquatic sediments. Naturally abundant in these environments, HS account for approximately 60–70% of the total organic matter. Their use has a long historical background across various cultures: in ancient Eastern medicine, humic-rich materials such as *Shilajit*—known as the “blood of the mountains”—were valued for their rejuvenating and restorative properties, while in ancient Egypt, similar substances were employed in preservation rituals [16].

Chemically, HS consist of large, polydisperse macromolecules (20,000–150,000 Da) derived primarily from lignite. Their polyaromatic backbone carries multiple functional side chains, including carboxyl, phenolic, quinone, hydroxyl, and amide groups, which confer both their biological activity and pH-dependent solubility—being soluble in alkaline but not acidic media [17].

Rich in phenolic, carboxylic, and quinone functional groups, HS exhibit redox buffering, metal chelation, and antioxidant capacities, alongside diverse biological activities [18]. In both veterinary and human nutrition, HS have been reported to confer multiple benefits through their effects on intestinal health and immune modulation. In humans, their role as dietary supplements with microbiota-modulating potential has been well characterized [19]. Through these functions, HA can neutralize ROS and beneficially modulate the gut microbiota by enhancing the abundance of *Lactobacillus* and *Bifidobacterium* while reducing *Proteobacteria* populations. Such prebiotic-like effects promote short-chain fatty acid (SCFA) synthesis—particularly butyrate—which fortifies epithelial TJs, lowers intestinal pH, and inhibits pathogenic bacterial proliferation [20].

Studies in livestock, including horses, pigs, ruminants, and poultry, have demonstrated that oral HA supplementation (500–2000 mg/kg body weight) effectively alleviates diarrhea, dysbiosis, and toxin-induced enteropathies. In poultry, HA supplementation altered gut microbiota composition, increased SCFA production, and improved trace element absorption [21]. Despite these benefits, the systemic bioavailability of HA remains extremely low (<0.1%), suggesting their primary action occurs locally within the gastrointestinal tract. Beyond their antioxidative properties, HA possess notable immunomodulatory activity. Sodium humate has been shown to alleviate LPS-induced intestinal injury in rodents by enhancing TJ protein and mucin expression, while suppressing TLR4/NF-κB signaling and NLRP3 inflammasome activation [22]. HA have been shown to protect mast cells and lymphocytes, stimulate macrophages and neutrophils, and suppress the secretion of proinflammatory cytokines such as TNF-α and IL-6 [23]. Mechanistic studies further reveal that HA inhibit the IκB kinase/NF-κB signaling pathway by preventing IκB degradation and NF-κB nuclear translocation, thereby attenuating the transcription of inflammatory genes [24]. Collectively, these findings highlight the multifaceted biological potential of HA; however, to date, the cellular effects of humic acid on epithelial barrier integrity and inflammatory responses in canine-relevant in vitro systems remain poorly characterized.

The aim of this study was to investigate the impact of a humic acid-based product on the gut-immune interplay with the use of two complementary in vitro models, specifically the IPEC-J2 cell line and peripheral blood mononuclear cells isolated from canine blood. The IPEC-J2 cell line is a well-characterized, non-transformed cell line of porcine jejunum origin [25]. It is a commonly employed tool for evaluating the effects of various feed additives [26,27,28,29,30]. Their physiological resemblance to canine enterocytes makes them highly suitable for generating cross-species translational insights [31]. Peripheral blood mononuclear cells (PBMCs) are easily isolated from peripheral blood and despite differing in activation status, phenotype and composition from cells found in intestinal tissue they are still a valuable model for studying the immune modulatory effects of feed bioactive compounds [29].

Our goals were to evaluated the effects of HA—with and without LPS-induced stress—on the barrier integrity of IPEC-J2, assessing epithelial barrier integrity by FITC-dextran (FD4) paracellular flux (TEER was used only as a pre-treatment confluence check) and to investigate HA’s ability to modulate LPS-induced cytokine secretion (TNF-α, IL-6) in canine PBMCs, elucidating mechanisms via TLR4/NF-κB and inflammasome pathways.

We hypothesized that HA would preserve epithelial barrier function and attenuate pro-inflammatory cytokine response in immune cells, supporting its potential as a nutraceutical adjuvant in canine chronic enteropathies.

## 2. Materials and Methods

### 2.1. Cell Lines and Culture Conditions

#### 2.1.1. Porcine Intestinal Epithelial Cell Line (IPEC-J2)

The IPEC-J2 cell line, derived from the jejunum of neonatal, colostrum-deprived piglets, was cultured in a 1:1 mixture of Dulbecco’s Modified Eagle’s Medium and Ham’s F-12 Nutrient Mixture (DMEM/F12; Merck, Darmstadt, Germany). The medium was supplemented with 5% fetal bovine serum (FBS EuroClone, Pero, Italy), insulin (5 µg/mL), transferrin (5 µg/mL), selenium (5 ng/mL), epidermal growth factor (EGF, 5 ng/mL), and penicillin-streptomycin (100 U/mL and 100 µg/mL, respectively, Lonza, Verviers, Belgium) [32]. For experimental treatments, plain (supplement-free) DMEM/F12 was used. Cells with passage numbers between 45 and 65 were seeded onto uncoated 96-well polystyrene culture plates at a density of approximately 1 × 10^4^ cells/well. Media were replaced every other day. For barrier integrity (paracellular permeability) assessments, cells were cultured on polyester membrane inserts (0.4 µm pore size, 12-well format) until a confluent monolayer formed, monitored via light microscopy and transepithelial resistance measurements.

#### 2.1.2. Canine Peripheral Blood Mononuclear Cells

Peripheral blood was collected from a clinically healthy adult dog under regular veterinary supervision, with written informed owner consent obtained prior to sampling. All experimental procedures complied with national and international regulations as well as institutional ethical standards. The study protocol was approved by the Food Chain Safety, Plant Protection and Soil Conservation Department of the Government Office of Pest County, Hungary (permit number: PE/EA/00980–6/2022).

PBMC isolation and culture conditions were performed according to established protocols based on density gradient centrifugation, as previously described for canine and human-derived PBMCs [12,29]. Whole blood samples were collected into sterile ethylenediaminetetraacetic acid (EDTA)-coated tubes and processed promptly after collection to minimize pre-analytical variability. Mononuclear cells were isolated using Histopaque 1077 (Sigma-Aldrich, Darmstadt, Germany) following the manufacturer’s instructions. Briefly, 3 mL of Histopaque 1077 was transferred into a 15 mL conical centrifuge tube and carefully overlaid with 3 mL of whole blood. Samples were centrifuged at 400× *g* for 30 min at 23 °C without brake. After centrifugation, the mononuclear cell layer located at the plasma–Histopaque interface was carefully aspirated and transferred into a new centrifuge tube. Cells were washed twice with 10 mL phosphate-buffered saline (PBS; Gibco, Paisley, UK) and once with 5 mL PBS, centrifuged at 250× *g* for 10 min at room temperature during each washing step, and the supernatant was discarded.

The final PBMC pellet was resuspended in RPMI-1640 culture medium (Merck, Darmstadt, Germany) containing L-glutamine and sodium bicarbonate, supplemented with 10% fetal bovine serum (FBS; EuroClone, Pero, Italy) and 1% penicillin–streptomycin solution (Lonza, Verviers, Belgium). The resulting PBMCs were washed twice with PBS, and cell viability was assessed using Trypan Blue exclusion (Sigma-Aldrich, Darmstadt, Germany) and a Bürker chamber. Cells were seeded at a density of 2 × 10^5^ cells/mL in 24-well plates using RPMI-1640 medium (Gibco, Paisley, UK) supplemented with 10% FBS, 1% PenStrep, L-glutamate, and sodium bicarbonate. Cultures were incubated overnight at 37 °C in 5% CO_2_ and 95% humidity.

### 2.2. Humic Acid Extract Product Preparation

The tested humic acid extract product was manufactured by Humac Hungary Kft (Tokaj, Hungary) under the trade name “Humac^®^ Natur AFM”. It is a natural, water-insoluble, activated-leonardite derivative with 66.6% total humic acid content (free humic acids ≥ 60%, fulvic acid ≥ 5%). Solubilization in organic solvents alters its bioactivity and is incompatible with cell culture systems. Therefore, based on consultation with the product developer and relevant literature [30], a preparation protocol was developed: the product was suspended in dimethyl sulfoxide (DMSO), subjected to two 10 min sonication cycles (37 kHz, room temperature; Elmasonic S, Singen, Germany), and then diluted in culture medium to a final concentration of 10 mg/mL (1% *v*/*v* DMSO). For PBMCs, the suspension was filtered (0.22 µm) prior to treatment. For IPEC-J2 cells, both sonicated and non-sonicated suspensions were applied without filtration, mimicking in vivo exposure via oral administration. Cells were incubated with treatment media for 24 or 48 h.

### 2.3. Inflammation Induction

Inflammation was induced using LPS derived from *Salmonella enterica* serovar Typhimurium and serovar Enteritidis (Sigma-Aldrich, Darmstadt, Germany). LPS was applied at a final concentration of 10 µg/mL, which was selected based on previous in vitro studies demonstrating robust and reproducible activation of pro-inflammatory signaling and cytokine release in PBMCs without compromising cell viability [23,33,34]. In preliminary experiments performed in our PBMC system, this concentration consistently induced TNF-α and IL-6 secretion over 24–48 h while maintaining metabolic activity, as confirmed by CCK-8 viability assays. Lower LPS concentrations (≤1 µg/mL) resulted in high inter-assay variability and insufficient cytokine induction, whereas higher concentrations did not further enhance the inflammatory response. LPS was added directly to PBMC cultures either alone or in combination with humic acid extract, and cells were incubated for 24 or 48 h. This exposure period was selected to capture both early and sustained inflammatory responses, consistent with previous reports investigating LPS-induced immune activation in canine and porcine-derived cell models [26].

### 2.4. Cell Viability Assay

The metabolic activity of both cell types was assessed using the Cell Counting Kit-8 (CCK-8; Sigma-Aldrich, Darmstadt, Germany), which quantifies the reduction in WST-8 to water-soluble formazan by cellular dehydrogenases. Absorbance was measured at 450 nm using a SpectraMax iD3 (Molecular Devices, San Jose, CA, USA) microplate reader.

### 2.5. Barrier Function (Paracellular Permeability) Assessment

The effects of humic acid and LPS on the paracellular permeability (barrier integrity) of IPEC-J2 cells were assessed using the tracer dye FD4 (Sigma-Aldrich, Darmstadt, Germany). Cells were seeded onto 12-well membrane inserts, and before applying the treatments, TEER measurements (using EVOM volt/ohm meter) were performed to confirm the formation of a differentiated, confluent monolayer. TEER measurements were used solely to verify formation of a confluent monolayer prior to initiating treatments; TEER was not evaluated as an experimental endpoint in this study. Following exposure to humic acid and/or LPS, cells were washed with PBS, and FD4 (dissolved in phenol-free DMEM/F12 medium) was added to the apical compartment at a final concentration of 0.25 mg/mL. Phenol-free DMEM/F12 medium was added to the basolateral chamber. The cells were then incubated at 37 °C with 5% CO_2_. After 24 h, 100 µL samples were collected from the basolateral compartment, and fluorescence was measured using a SpectraMax iD3 instrument with 485 nm excitation and 535 nm emission wavelengths. The 24 h FD4 collection period was selected based on previous experiments by our research group (2 h and 4 h FD4 exposure), where no significant group differences were detectable, indicating that a longer accumulation period was required to obtain sufficient sensitivity in this model. Results are therefore interpreted as cumulative FD4 transfer over 24 h [35].

### 2.6. Measurement of Intracellular ROS Levels

Intracellular ROS levels were assessed using the DCFH-DA assay, a widely applied method for detecting oxidative stress in immune and epithelial cells, as previously described [26,36]. DCFH-DA, a non-fluorescent precursor, readily permeates the cell membrane, where it is deacetylated by intracellular esterases to form the non-fluorescent compound dichlorofluorescin (DCFH). Elevated intracellular ROS oxidize DCFH to the highly fluorescent dichlorofluorescein (DCF). Thus, the fluorescence intensity is directly proportional to the intracellular ROS level. Following experimental treatments, cells were incubated with 10 µM DCFH-DA in phenol red-free culture medium for 60 min at 37 °C in the dark. After incubation, the dye-containing medium was removed, and cells were washed twice with PBS to eliminate extracellular probe. Cells were then lysed using M-PER Mammalian Protein Extraction Reagent (Thermo Fisher Scientific, Waltham, MA, USA), scraped, and collected into microtubes. Lysates were centrifuged at 1000× *g* for 10 min at 4 °C to remove cellular debris. Fluorescence was measured from clarified cell lysates, reflecting intracellular DCF generated within cells using a SpectraMax iD3 microplate reader (Molecular Devices, San Jose, CA, USA) at excitation and emission wavelengths of 485 nm and 535 nm, respectively. Fluorescence values were expressed relative to untreated controls.

### 2.7. Cytokine Quantification in PBMC Supernatants

Concentrations of TNF-α and IL-6 in PBMC supernatants were quantified using canine-specific ELISA kits (Merck, Darmstadt, Germany) according to the manufacturer’s instructions, following 24 and 48 h treatments with LPS and/or humic acid extract. Absorbance was measured at 450 nm using a SpectraMax ID3 Microplate Reader (Molecular Devices, San Jose, CA, USA). Cytokine concentrations were calculated from standard calibration curves generated from known concentrations of recombinant canine TNF-α and IL-6 standards, and data were expressed as pg/mL. All samples and standards were measured in duplicate to ensure analytical accuracy.

### 2.8. Statistical Analysis

All experiments were performed with six independent culture replicates (wells) per treatment and time point. Data were analyzed using R software (version 3.3.2; R Foundation for Statistical Computing, Vienna, Austria). Prior to analysis, datasets were screened for outliers using Grubbs’ test, and data distribution was assessed for normality using the Shapiro–Wilk test. Homogeneity of variances was evaluated using Levene’s test. For statistical comparisons, one-way analysis of variance (ANOVA) was performed separately for each time point, followed by Tukey’s post hoc test to identify significant pairwise differences among treatment groups. Measurements at 24 h and 48 h were obtained from separate, independently cultured wells, and therefore were treated as statistically independent observations rather than repeated measures. Results are expressed as mean ± standard error of the mean (SEM), and statistical significance was accepted at *p* ≤ 0.05.

## 3. Results

### 3.1. Effect of Humic Acid Extract on Cell Viability

No significant differences in metabolic activity were observed in PBMC cultures following 24 or 48 h of humic acid extract treatment compared to controls. Similarly, IPEC-J2 cells treated with both sonicated and non-sonicated humic acid extract suspensions exhibited no significant reductions in viability at either time point. These findings confirm that the tested concentration of Humic acid extract (10 mg/mL) does not exert cytotoxic effects under the applied conditions.

### 3.2. Effect of Humic Acid Extract on Intestinal Barrier Integrity

**Figure 1:**
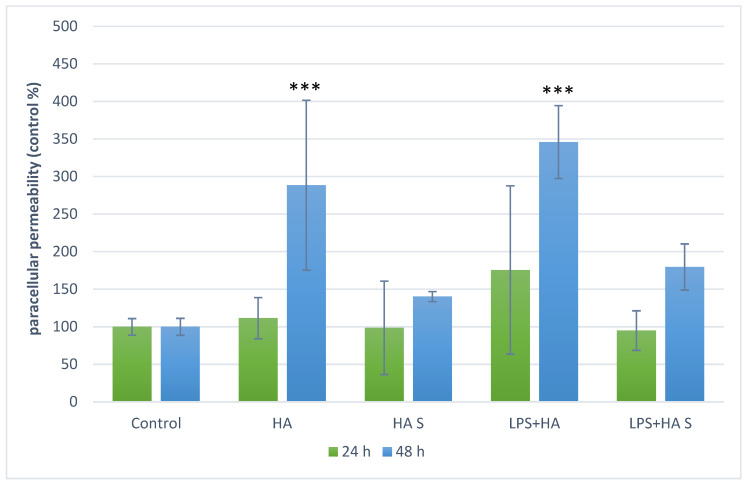
Paracellular permeability of IPEC-J2 cell monolayers following humic acid extract and LPS treatments; green bars represent results after 24 h, blue bars after 48 h; *** *p* < 0.001 compared to control group. Treatment labels: Control—cell culture medium, HA—humic acid extract 10 mg/mL, HA S—humic acid extract 10 mg/mL sonicated, LPS—lipopolysaccharide from *Salmonella enterica* serovar Typhimurium 10 µg/mL, LPS+HA—humic acid extract 10 mg/mL combined with *Salmonella enterica* serovar Typhimurium LPS 10 µg/mL, LPS+HA S—sonicated humic acid extract 10 mg/mL combined with *Salmonella enterica* serovar Typhimurium LPS 10 µg/mL.

As shown in Figure 1, barrier integrity (FD4 flux) differed depending on humic acid extract preparation and exposure duration. Non-sonicated humic acid extract increased FD4 translocation after 48 h, consistent with reduced monolayer barrier integrity under these conditions. In contrast, the sonicated suspension did not increase FD4 flux at either time point. In the co-exposure conditions tested (humic acid extract with LPS), a similar pattern was observed: FD4 flux remained lower in the sonicated humic acid extract group than in the corresponding non-sonicated group.

### 3.3. Measurement of Intracellular ROS Levels

**Figure 2:**
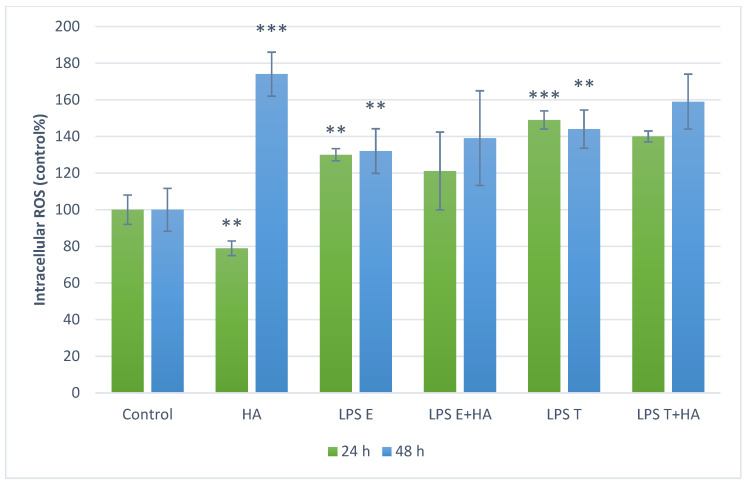
Intracellular reactive oxygen species (ROS) levels in canine peripheral blood mononuclear cell (PBMC) cultures following LPS and humic acid extract treatments; green bars represent results after 24 h, blue bars after 48 h; values are expressed as percentages of the control group; ** *p* < 0.01, *** *p* < 0.001 compared to control group. Treatment labels: Control—cell culture medium; HA—humic acid extract 10 mg/mL; LPS E—lipopolysaccharide from *Salmonella enterica* serovar Enteritidis 10 µg/mL; LPS T—lipopolysaccharide from *Salmonella enterica* serovar Typhimurium 10 µg/mL; LPS E+HA—*Salmonella enterica* serovar Enteritidis LPS 10 µg/mL combined with humic acid extract 10 mg/mL; LPS T+HA—*Salmonella enterica* serovar Typhimurium LPS 10 µg/mL combined with humic acid extract 10 mg/mL.

Intracellular ROS levels were measured in PBMC cultures using the DCF assay, and results were expressed as a percentage of the control. Treatment with humic acid extract alone reduced ROS production after 24 h but significantly increased it after 48 h. Both *S. enterica* serovar Enteritidis (LPS E) and *S. enterica* serovar Typhimurium (LPS T) stimulation markedly increased ROS levels at both 24 and 48 h. Co-treatment with humic acid extract did not significantly modify the ROS response induced by either LPS preparation (Figure 2).

### 3.4. Anti-Inflammatory Effect of Humic Acid Extract on PBMC Cultures

**Figure 3:**
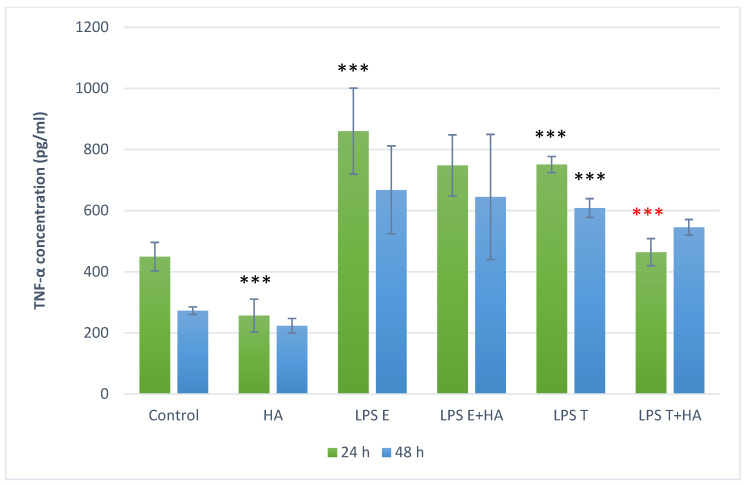
TNF-α concentration in canine peripheral blood mononuclear cell (PBMC) cultures following LPS and Humic acid extract treatments; green bars represent results after 24 h, blue bars after 48 h; *** *p* < 0.001 compared to control group, *** *p* < 0.001 compared to LPS treatment. Treatment labels: Control—cell culture medium, HA—humic acid extract 10 mg/mL, LPS E—lipopolysaccharide from *Salmonella enterica* serovar Enteritidis 10 µg/mL, LPS T—lipopolysaccharide from *Salmonella enterica* serovar Typhimurium 10 µg/mL, LPS E+HA—*Salmonella enterica* serovar Enteritidis LPS 10 µg/mL combined with humic acid extract 10 mg/mL, LPS T+HA—*Salmonella enterica* serovar Typhimurium LPS 10 µg/mL combined with humic acid extract 10 mg/mL.

Cytokine analysis (Figure 3) revealed that humic acid extract alone significantly reduced TNF-α levels after 24 h (*p* < 0.01), with no pro-inflammatory effects observed. LPS stimulation with *S. enterica* ser. Enteritidis significantly increased TNF-α production (*p* < 0.001 at 24 h) and *S. enterica* ser. Typhimurium significantly increased TNF-α production (*p* < 0.001 at 24 h and at 48 h). Co-treatment with humic acid extract markedly attenuated this response (*p* < 0.001 at 24 h), but the effect diminished by 48 h (*p* = 0.826). In contrast, no significant anti-inflammatory effect was observed for *S. enterica* ser. Enteritidis-induced inflammation.

**Figure 4:**
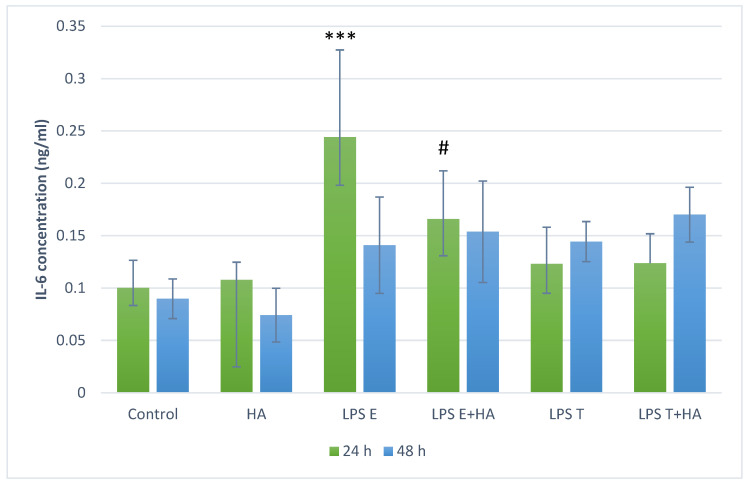
IL-6 concentration in canine peripheral blood mononuclear cell (PBMC) cultures following LPS and humic acid extract treatments; green bars represent results after 24 h, blue bars after 48 h; *** *p* < 0.001 compared to control group, # *p* < 0.1 compared to LPS treatment. Treatment labels: Control—cell culture medium, HA—humic acid extract 10 mg/mL, LPS E—lipopolysaccharide from *Salmonella enterica* serovar Enteritidis 10 µg/mL, LPS T—lipopolysaccharide from *Salmonella enterica* serovar Typhimurium 10 µg/mL, LPS E+HA—*Salmonella enterica* serovar Enteritidis LPS 10 µg/mL combined with humic acid extract 10 mg/mL, LPS T+HA—*Salmonella enterica* serovar Typhimurium LPS 10 µg/mL combined with humic acid extract 10 mg/mL.

In IL-6 assays (Figure 4), Enteritidis-derived LPS induced a significant increase in IL-6 levels at 24 h (*p* < 0.001). Co-treatment with humic acid extract led to a modest reduction in IL-6 concentration (*p* < 0.1), while no effects were observed at 48 h.

## 4. Discussion

Chronic inflammatory enteropathies affect a large number of dogs worldwide. Although the disease is multifactorial, maintaining intestinal barrier integrity and a well-regulated immune system is of paramount importance—both of which can potentially be supported through supplementation with HS–containing products. The intestinal barrier plays a crucial role in preventing the entry of harmful substances, while balanced oxidative status, immune homeostasis, and a diverse microbiota are key components of gut health [31,37]. HA have been reported to beneficially influence these parameters, yet the exact mechanisms through which HAs act on intestinal epithelial and PBMCs in dogs remain poorly characterized. In the first phase of our study, we examined whether the applied humic acid concentration (10 mg/mL), preparation method (sonicated vs. non-sonicated), and treatment duration (24 vs. 48 h) affected the metabolic activity of IPEC-J2 and canine PBMCs. No cytotoxic effect was observed under any condition, which aligns with previous studies reporting no HA-induced cytotoxicity in human PBMCs or IPEC-J2 cells [38,39]. These findings helped us establish the optimal conditions for subsequent experiments. Previous studies have shown that intestinal barrier dysfunction is a hallmark of enteropathies [40]. LPS has been reported to impair tight junction integrity in intestinal epithelial models [22,41]. This was also confirmed by our research group previously [35]. In our study, we evaluated FD4 flux following exposure to humic acid preparations in the presence of LPS as a stressor; however, because an epithelial LPS-only group was not included, we do not make causal conclusions about LPS-specific barrier disruption in this dataset. Rather, we interpret the permeability results as differences among the tested treatment combinations and highlight the need for an epithelial LPS-only control in future experiments. Because an epithelial LPS-only control was not included, we refrain from attributing changes specifically to LPS and interpret these data as group-wise differences among the tested conditions. Because FD4 was collected over 24 h (cumulative flux), this readout is interpreted as an integrated permeability endpoint rather than an instantaneous permeability coefficient. These results are consistent with reports that HA can restore LPS-induced barrier injury [21]. Interestingly, prolonged (48 h) treatment with non-sonicated HA increased paracellular permeability, suggesting that barrier integrity was not restored. The same pattern emerged when HA was applied alone, indicating a possible time-dependent shift in HA behavior. ROS generation can damage TJ proteins, leading to increased paracellular permeability. Several studies have demonstrated both prooxidant and antioxidant properties of HA—depending on their structure and environmental conditions [42,43]. The solubility of HA is highly pH-dependent [23]; thus, extended incubation (48 h) may have altered the medium’s pH, creating conditions favoring the dissolution of prooxidant compounds. Differences observed between sonicated and non-sonicated treatments can likely be attributed to particle aggregation and membrane interactions. Humic acids form large, heterogeneous aggregates in solution, which can mechanically disrupt epithelial layers or interfere with TJ organization. Sonication disperses these aggregates into smaller, more uniform particles, improving solubility and reducing their disruptive potential [44,45]. Time-dependent changes in aggregation behavior may further explain the observed effects, though this requires confirmation using analytical methods optimized for measuring particle aggregation. Comparing HA effects across studies remains challenging due to the complex and variable composition of humic materials. Two HA preparations from different natural sources rarely share identical molecular profiles—some may exert protective effects, whereas others can be neutral or even detrimental [30]. Considering the physicochemical variability of HA and the gastrointestinal transit time in dogs, a 24 h treatment window appears most appropriate for modeling intestinal effects in vitro. Potential interaction between particulate humic material and fluorescence-based readouts is a limitation and will be addressed in future work using acellular spike-recovery controls. Another limitation of the epithelial permeability experiment is the absence of an epithelial LPS-only control, which precludes direct attribution of permeability changes to LPS.

Barrier dysfunction can permit translocation of luminal bacterial components such as LPS, which can activate immune responses. While systemic consequences of endotoxemia have been discussed in the broader literature [46], our study did not measure circulating endotoxin or systemic outcomes. Therefore, we restrict interpretation to our in vitro endpoints in epithelial cells (FD4 flux) and PBMC responses (ROS and cytokine production), and present systemic considerations only as contextual background. If luminal LPS translocates into the circulation, metabolic endotoxaemia triggers oxidative stress and a state of chronic low-grade inflammation via activation of receptors such as Toll-like receptor 4 and downstream NADPH oxidase pathways. Such persistent inflammation and oxidative damage are strongly linked to metabolic syndrome, insulin resistance, non-alcoholic fatty liver disease and cardiovascular dysfunction. In addition, emerging evidence suggests an association between endotoxaemia and neurological disorders—via barrier disruption, neuroinflammation and oxidative injury [47]. Thus loss of mucosal barrier function and subsequent LPS translocation can instigate a cascade of systemic adverse effects well beyond the gut itself. Therefore, in the next experimental phase, we used whole blood from healthy dogs to model systemic endotoxin exposure—a key feature of CIEs. In LPS-stimulated PBMCs oxidative stress and inflammatory responses were investigated. In our oxidative stress assays, HA treatment alone reduced ROS levels after 24 h but increased them after 48 h, suggesting a possible time-dependent prooxidant effect. When co-applied with LPS, HA did not significantly alter ROS levels, implying that its antioxidant effect may be preventive rather than therapeutic once oxidative stress is established. This biphasic (antioxidant/prooxidant) behavior has been documented previously [22]. HA significantly reduced TNF-α and IL-6 production after 24 h, while effects after 48 h were less pronounced. These results are consistent with previous in vitro studies showing NF-κB–mediated inflammatory suppression by humic substances in human and rodent models [47,48]. Humic acid has been shown to inhibit LPS-induced NF-κB activation and adhesion molecule expression in human endothelial cells [49], as well as dose-dependent suppression of TNF-α and IL-6 in macrophage-like U937 cells [23]. Sodium humate also alleviated LPS-induced liver injury in mice by suppressing the TLR4/NF-κB inflammatory axis and activating the NRF2/HO-1 antioxidant pathway [49]. Similarly, in murine models of dextran sulfate sodium-induced colitis, humic acids mitigated inflammation, preserved mucosal integrity, and inhibited pathogen proliferation [20]. In vivo, dietary HA reduced endotoxin-induced inflammation in piglets, enhancing immune resilience [50]. Overall, HA (particularly in sonicated form) preserved epithelial barrier integrity in the IPEC-J2 model and reduced pro-inflammatory cytokine (TNF-α and IL-6) production in LPS-stimulated canine PBMCs. These effects align with the concept that barrier dysfunction and cytokine-driven inflammation contribute to chronic enteropathies in dogs [51]. While canine-specific mechanistic data remain limited, our findings support further targeted studies of HA as a nutraceutical candidate for barrier support and inflammatory modulation.

The present study has several limitations that should be considered when interpreting the results. First, the in vitro models applied—namely IPEC-J2 intestinal epithelial cells and canine PBMCs—do not fully recapitulate the complexity of the canine intestinal environment, which involves dynamic interactions between epithelial cells, immune cells, microbiota, and luminal factors. In particular, the lack of a microbial component precluded assessment of humic acid–mediated effects on microbial fermentation and short-chain fatty acid production. Although IPEC-J2 cells originate from porcine jejunum, they are widely used as a translational model for studying intestinal barrier function and innate immune responses, and they exhibit tight junction organization and epithelial physiology comparable to canine enterocytes. Nevertheless, species-specific differences cannot be excluded and should be considered when extrapolating the findings to dogs. Another limitation of the study is that mechanistic signaling pathways were not directly assessed. While the observed effects of humic acid on barrier integrity and cytokine production are consistent with previously reported modulation of TLR4/NF-κB–related pathways, direct evaluation of pathway activation—such as NF-κB nuclear translocation or inflammasome signaling—was beyond the scope of the present work. In addition, barrier function was evaluated using cumulative FD4 flux over a 24 h period rather than instantaneous permeability coefficients. While this approach provided sufficient sensitivity to detect treatment-related differences, potential interactions between particulate humic material and fluorescence-based readouts cannot be fully excluded. Future studies incorporating acellular controls and complementary permeability assays are therefore warranted. Finally, PBMCs primarily represent systemic immune responses and may not fully reflect the behavior of mucosal immune cells residing in the intestinal lamina propria. Accordingly, the immunomodulatory effects observed here should be interpreted as indicative of systemic inflammatory modulation rather than direct mucosal immune regulation. PBMC experiments were performed using cells isolated from a single donor dog; therefore, the results reflect within-donor treatment effects and should be interpreted with this limitation in mind.

## 5. Conclusions

In conclusion, the present study demonstrates that HA can exert protective effects on intestinal barrier integrity and modulate inflammatory cytokine responses in cell-based models relevant to canine gut health. Based on experiments using IPEC-J2 epithelial cells and canine PBMCs, appropriately processed humic acid preparations were shown to preserve epithelial barrier function and attenuate LPS-induced pro-inflammatory signaling under in vitro conditions. These findings support the concept that HA may act through a dual mechanism, reinforcing epithelial defenses locally while modulating systemic immune activation. However, given the inherent limitations of the applied in vitro models, the results should be regarded as proof-of-concept evidence rather than direct predictors of in vivo efficacy. Future studies employing physiologically more complex systems, such as canine intestinal organoids, epithelial–immune co-culture models, and in vivo investigations in dogs, will be essential to confirm the translational relevance of these effects. Together, the present data provide a rational experimental basis for further evaluation of humic acid extracts as nutraceutical adjuncts in the management of canine chronic enteropathies.

## Data Availability

All data that support the above-detailed findings can be obtained from the corresponding author upon request.
